# What can artificial intelligence bring to Alzheimer’s disease clinical trials? A first perspective

**DOI:** 10.1016/j.tjpad.2025.100423

**Published:** 2025-12-01

**Authors:** Lefkos T Middleton, Sandrine Andrieu

**Affiliations:** a Imperial College London, London, United Kingdom; b IHU HealthAge, Toulouse III University-Paul Sabatier, INSERM CERPOP, Toulouse, France

Recent pharmaceutical advances and the development of sensitive and specific blood-based biomarkers are reinvigorating the field of Alzheimer’s disease (AD) and related dementias (ADRD). The pharmaceutical research and development pipeline is expanding rapidly, encompassing both traditional targets, namely, the amyloid and tau pathways of AD, and a growing array of candidate pathways and novel mechanisms [1].

Concurrently, the repertoire of blood-based biomarkers continues to evolve. Emerging from diverse AT[N] assay platforms and both targeted and untargeted multi-omics technologies, these biomarkers hold the promise of enhancing the accuracy and precision with which the biological and clinical underpinnings of ADRD—and their potential subtypes—are characterized. Such progress represents an essential step towards the realization of evidence-based precision medicine. These developments pave the way for innovative preventative and therapeutic strategies, including combination therapies that have already demonstrated substantial benefit in the HIV/AIDS pandemic, as well as cancer and other complex, multifactorial diseases.

In parallel, several international data-sharing initiatives, such as the Global Neurodegeneration Proteomics Consortium, exemplify the commitment of key observational and interventional cohort studies to harmonize and make state-of-the-art datasets accessible to the broader scientific community. Such collaborations are critical to accelerating discovery and translation in the ongoing effort to address the significant challenges posed by ADRD and other neurodegenerative diseases.

At this juncture, there is increasing recognition across the AD research and clinical community—including academia, industry, and healthcare—of the transformative, multi-dimensional potential of artificial intelligence (AI) in discovery research and clinical development. AI offers powerful tools to enhance literature review processes, facilitate data harmonization, extract meaningful insights from high-dimensional digital and biomarker data, and aid in data interpretation. Moreover, AI holds promise in optimizing patient stratification and accelerating recruitment within clinical trials. These topics, alongside a critical appraisal of the potential risks and limitations of AI applications, are addressed throughout this special issue.

With these considerations in mind, we would like to express, on behalf of the Editorial Board, our sincere gratitude to the authors who have contributed to this special issue. Their diverse yet complementary perspectives provide valuable insights into how AI can advance disease understanding, refine diagnostic precision, and enable the development of effective preventative and therapeutic interventions. Collectively, these contributions mark a significant step forward as we enter a new era of research and clinical innovation in Alzheimer’s disease and related dementias.

The Editors are in agreement with the view expressed by Moore [2] et al. that “AI is not an autonomous solution, but rather a powerful amplifier and accelerator of human expertise and its greatest value will come from fusing computational power with the insight, creativity, and compassion of the scientific and medical community”.

## Declaration of competing interest

The authors declare that they have no known competing financial interests or personal relationships that could have appeared to influence the work reported in this paper.
